# Linking high-sensitivity C-reactive protein and dyslipidemia among patients with type 2 diabetes mellitus

**DOI:** 10.6026/973206300211357

**Published:** 2025-06-30

**Authors:** Ramesh Dnyandeo Kurle, Veluri Ganesh, Dileep Khubya, Gadigeppa H Chittaragi

**Affiliations:** 1 Department of General Medicine, Dr. N Y Tasgaonkar Institute of Medical Science, Diksal, Maharashtra, India; 2 Department of Biochemistry, PES University Institute of Medical Sciences and Research, Electronic City, Bengaluru, Karnataka, India; 3 Department of Biochemistry, Sri Siddhartha Institute of Medical Sciences, T Begur, Nelamangala, Karnataka, India; 4 Department of Medicine, Dr. N Y Tasgaonkar Institute of Medical Science, Diksal, Maharashtra, India

**Keywords:** High sensitivity-C-reactive protein (hs-CRP), hypertension, type 2 diabetes mellitus, myocardial infarction

## Abstract

Type 2 diabetes is evaluated in a condition elevated hs-CRP is linked to an increased risk of myocardial infraction development. Therefore, it is of interest to evaluate the hs-CRP in patients with type 2 diabetes mellitus who were not diagnosed with hypertension. We included total 120 T2DM patients attended to general medicine department and 60 healthy controls also included in the case control study. We measured demographic, anthropometric and clinical parameters from all the subjects. The type 2 diabetes mellitus patients with hypertension who had increased hs-CRP levels had a greater incidence of myocardial infraction results death and morbidity when compared to type 2 diabetes mellitus patients without hypertension. We show that CRP is a strong predictor of hospital morbidity and mortality in patients with acute myocardial infarction who have diabetes with hypertension as well as those who do not have hypertension.

## Background:

Type 2 Diabetes Mellitus (T2DM) is a major global public health concern due to its high incidence and consequences [1]. Metabolic changes that link hyperglycemia to other risk factors that lead to circulatory system problems, like high blood pressure (HBP), are the disease's defining feature [2-3]. With four million instances annually, T2DM related deaths account for 9% of all deaths worldwide. There are four million instances of T2DM annually, accounting for 9% of all deaths worldwide [4-5]. In T2DM patient's cardiovascular diseases (CVD), which include peripheral arterial obstructive disease, ischemic stroke, myocardial infarction, and various other associated conditions, are the leading cause of mortality [6- 7]. As of right now, atherosclerosis is thought to be the primary cause of CVD development. The primary theory on the genesis of atherosclerosis holds that it is an inflammatory process that can take many different forms and damages the arterial endothelium [8]. Prolonged inflammation promotes the clinical development of atheroma plaques, which can burst and cause thrombus formation and other atherosclerosis-related problems [9]. The high-sensitivity C-reactive protein (hs-CRP) a 21,500 Da typical acute phase inflammation response protein, rise in inflammatory and infected circumstances. Mostly produced in the liver by inducing inflammatory cytokines like interleukin-6, it has a half-life of up to 19 hours [10-11]. The previous research has found a correlation between hs-CRP levels and factors such as race, body mass index (BMI), income, education, insurance status, disability status, smoking, high blood pressure and diabetes [12-13]. The recent investigations have shown that inflammation is a significant factor in the development of cardiovascular illnesses and arteriosclerosis in T2DM Patients [14-15]. Increased levels of hs-CRP, a measure for systemic inflammation, are linked to future cardiovascular risk, according to numerous epidemiological and intervention studies. Nevertheless, at this point, measuring hs-CRP may be helpful in determining a patient's risk of complications from diabetes [16]. Furthermore, there was a association between hypertension and hs-CRP has only been examined in a few number of research; nevertheless, those that have been conducted and demonstrated that hypertensive individuals had greater levels of hs-CRP than normotensive participants. According to the majority of clinical research, CRP is a stand-alone indicator for predicting the risk of atherosclerosis, myocardial infarction, hypertension, and cardiovascular events [17- 18]. Therefore, it is of interest to evaluate the correlation between high-sensitivity C-reactive protein with dyslipidemia in patients with type 2 diabetes mellitus.

## Materials and Methods:

One hundred twenty with T2DM patients in the Dr. N Y Tasgaonkar Institute of Medical Science, Maharashtra PES University Institute of Medical Sciences and Research, Karnataka for the period from May 2023 to March 2025. The T2DM Patients further subgroup into two groups based on the status of hypertension; sixty (60) T2DM patients without hypertension (group-2) and remaining sixty (60) T2DM with hypertension (Group-3). Additionally, we included 60 age, gender matched healthy volunteers consider as controls (Group-1). The study was done after taken approval from Institutional Ethics Committee (IEC) and the study participants were recruited after obtained informed consent forms.

## Criteria of the study:

## Inclusion criteria:

Patients who are older than thirty and have been diagnosed with type 2 diabetes mellitus with and without hypertension. The controls should not have any illness. Exclusion criteria: The subjects have history of smoking, alcoholism, Non-Diabetic Renal Disease, Urinary Tract Infections, Type 1 Diabetes Mellitus, People taking anti-inflammatory, immunosuppressive, and thiazolidinedione medications, Liver and thyroid disorders, Macro vascular issues like peripheral vascular disorders and cerebrovascular disorders, Inflammatory disease that is active and unwilling to take part were excluded from the study.

## Specimen collection:

All subjects got five (5) millilitres of fasting venous blood drawn into three tubes: one milliliter into an anti-glycolytic tube, one milliliter into an Ethylene Diamine Tetra Acetic acid (EDTA) and three millilitres into a plain tube. The plain samples were allowed to coagulate and then separated by centrifugation at 3000 rpm for 15 minutes, while plasma samples were separated right away. After being separated, the samples were placed in aliquots with the proper labels and kept at -800C until biochemical analysis was completed.

## Analysis:

The blood sugars, lipid profile, hs-CRP determined by international federation of clinical chemistry methods. The glycated hemoglobin was analyzed by using high performance liquid chromatography method.

## Statistical analysis:

To determine if the data was normally distributed, the Kolmogorov Smirnov test was used. Mean ± standard deviation or median (interquartile range) were used to express the data for regularly and non-normally distributed data, respectively. Analysis of variance (ANOVA) test was used to compare the hs-CRP and other study parameters levels of T2DM patients with and without hypertension to controls. Analysis of the Pearson correlations was used to examine the relationship between the variables. The spread sheets in Microsoft Excel and SPSS for Windows version 16.0 were used for statistical analysis. A p-value of less than 0.05 was regarded as statistically remarkable.

## Results:

The demographic traits and biochemical markers of type 2 diabetes mellitus patients and healthy controls are displayed in Table 1 (see PDF). When age was matched between the two groups, type 2 diabetes mellitus patients were significantly more than healthy controls (p<0.001). When compared to controls, type 2 diabetes mellitus patients showed significantly higher levels of BMI, SBP, DBP, FBS, PPBS, HbA1c, total cholesterol and triglycerides, VLDL and LDL (p=0.001**, respectively). Additionally, there was a significantly decreased level of HDL in type 2 diabetes mellitus patients and healthy controls. The hs-CRP and troponin levels were significantly increased in type 2 diabetes mellitus patients and healthy controls (p=0.001**). The demographic traits and biochemical markers both the groups of type 2 diabetes mellitus patients and healthy controls are displayed in Table 2 (see PDF). When age was matched between the two groups, type 2 diabetes mellitus patients were significantly more than healthy controls (p<0.001). When compared to controls, both the groups of type 2 diabetes mellitus patients showed significantly higher levels of BMI, SBP, DBP, FBS, PPBS, HbA1c, total cholesterol and triglycerides, VLDL and LDL (p=0.001**, respectively). Additionally, there was a significantly decreased level of HDL in both the groups of type 2 diabetes mellitus patients and healthy controls. The hs-CRP and troponin levels were significantly increased in both the groups of type 2 diabetes mellitus patients and healthy controls (p=0.001**). The correlations of hs-CRP with other parameters of study are displayed in Table 3 (see PDF). There was a significant positive correlation between hs-CRP and other parameters of the study (p=0.001**). Additionally, the hs-CRP negatively correlated with HDL respectively p=0.001**.

## Discussion:

Patients with Type 2 diabetes have higher rates of each kind of CVD, including prevalence, incidence, and mortality. As for the connection between inflammation and clinical outcomes, our study found that hs-CRP was linked to distinct outcomes in individuals with and without diabetes mellitus [19-20]. This could be because DM is a multifactorial metabolic disease that is marked by a state of subclinical inflammation. Chronically high levels of hs-CRP also indicate the more common association between diabetes mellitus and some degree of chronic inflammation. In general, atherosclerosis and type 2 - diabetes are complex conditions that may share an inflammatory foundation [21]. As a significant inflammatory marker, hs-CRP measurement can predict future vascular events on its own, improving the overall risk classification regardless of an individual's LDL-C values. The nature of systemic progressing atherosclerosis disease was linked to an elevated hs-CRP in a carotid artery risk for atherosclerosis study, which demonstrated that patients with increased inflammation are often at high risk for atherosclerosis progression [22]. One of the early markers to identify the patients' underlying subclinical inflammation was hs-CRP. In both patients with and without diabetes, it is a recognized indicator for predicting atherosclerosis and the problems associated with the disease [23]. It has been reported that CAD patients have higher hs-CRP concentrations than non-CAD patients. Hepatocytes and certain extra-hepatic tissues, including vascular smooth muscle, atherosclerotic plaques, and intracardial tissue, manufacture CRP, a member of the pentraxin protein family. Since cardiac imaging techniques rarely assess vascular inflammatory changes, testing for inflammation biomarkers in peripheral blood is becoming more important [24-26]. Arterial inflammation has been identified as a key element in the development and advancement of atherosclerosis, while systemic hypertension and diabetes mellitus are well-known risk factors for the disease [27]. In this investigation, T2DM and hypertension patients were positively and significantly correlated with elevated plasma hs-CRP levels. However, for the disorders that were identified, correlation was shown [28]. The plasma hs-CRP levels were substantially greater in hypertensive patients with type 2 diabetes mellitus than in normal participants, according to an assessment of a homogeneous population based on age. The hs-CRP is produced as a result of inflammation caused by a rise in advanced glycation end products, which may activate macrophages and enhance oxidative stress [29, 30- 31]. It has not been established that patients with T2DM have chronic low-grade inflammation linked to the development of atherosclerosis. Similarly, we also discovered that blood hs-CRP levels and age were reliable indicators of silent myocardial ischemia in diabetic patients. Prior studies have linked the risk of cardiovascular disease (CVD) to serum levels of hs-CRP, a measure of systemic inflammation and an atherosclerotic mediator. The based on study findings the significant elevated levels of hs-CRP positively correlated with dyslipidemia and cardiac troponins. Thus, the continuous monitoring of hs-CRP might be use full for early prediction of myocardial infraction in patients T2DM.

## Conclusion:

The circulating inflammatory marker hs-CRP levels were shown to be greater in type 2 diabetes mellitus with hypertensive when compared to type 2 diabetes mellitus without hypertensive and healthy controls. Thus the continuous monitoring of hs-CRP might be use full for early prediction of myocardial infraction in patients T2DM.
